# Treatment options of complicated urinary tract infections in ectopic kidneys: A case report

**DOI:** 10.1002/ccr3.1765

**Published:** 2018-09-04

**Authors:** Spyridon Triantafyllou, Stavros Aggelis, Dimitrios Tzavelas, Ifigeneia‐Vasiliki Kontoteza, Panagiotis Skandalakis, Dimitrios Filippou

**Affiliations:** ^1^ Department of Urology Athens General Hospital Hippocration Athens Greece; ^2^ Department of Anatomy and Surgical Anatomy Medical School National and Kapodestrian University of Athens Athens Greece

**Keywords:** ectopic kidney, pelvic ectopia, renal ectopia, urinary infections

## Abstract

Renal ectopia is a relatively rare situation which may complicate the diagnosis. In cases with renal ectopia, the normal route of the urinary tract alters and may be associated with increased incidence of infections or coexist with other malformations. We report a female patient with unobstructed pelvic renal ectopia complicated by urinary tract infection. After presenting our diagnostic and therapeutic dilemmas and strategy, we discuss the existing diagnostic and treating options including conservative and surgical approaches.

## 1 INTRODUCTION

Renal ectopia is the condition where kidney fails to reach its normal location in the renal fossa. The actual incidence varies among several studies from 1:500 to 1:1200 with an average occurrence of 1:900 with no significant difference between sexes.^1^, ^2^, ^3^ In most cases of renal ectopia is usually asymptomatic. However, nonspecific symptoms including abdominal discomfort, ureteric colic, or symptoms of urinary tract infection may contribute to set the diagnosis of ectopia. Ectopic kidneys are no more susceptible to disease than normal with the exception of hydronephrosis development, renal stones formation, and urinary tract infections.^4^ Aim of the current case report was to present the possible diagnostic and therapeutic options in urinary tract infections complicated by an ectopic kidney.

## 2 CASE PRESENTATION

**Figure 1:**
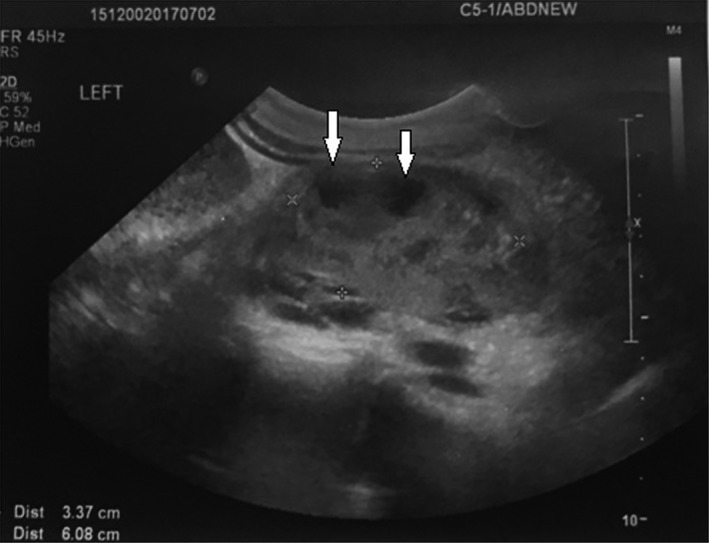
Ultrasound of the left pelvic ectopic kidney revealed multiple abscesses in its upper pole. The arrows in the figure indicate the locations of some them

**Figure 2:**
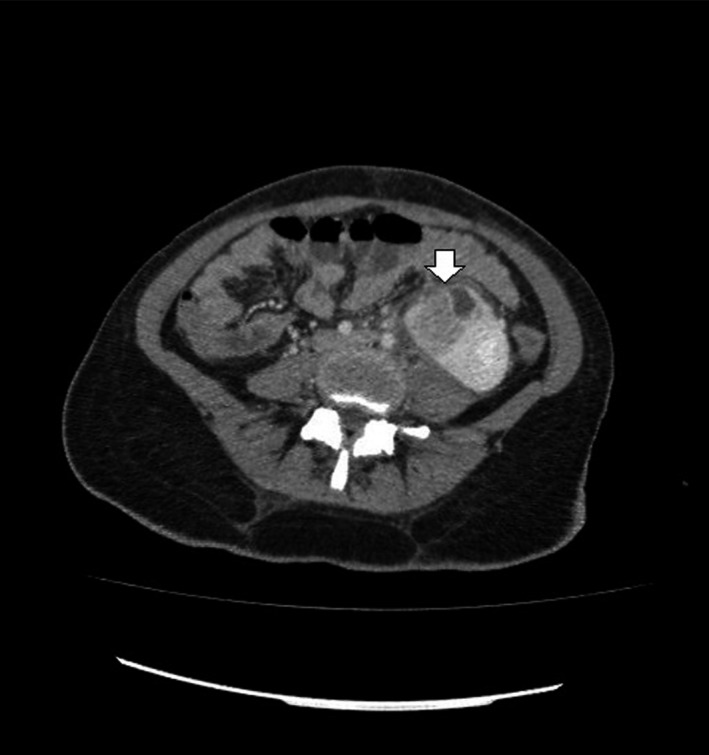
The abdominal CT scan which was performed after the initial assessment of the patient with the US confirmed the diagnosis of the left pelvic renal ectopia and the abscess formation in its upper pole (The arrow indicates the site of the abscess)

**Figure 3:**
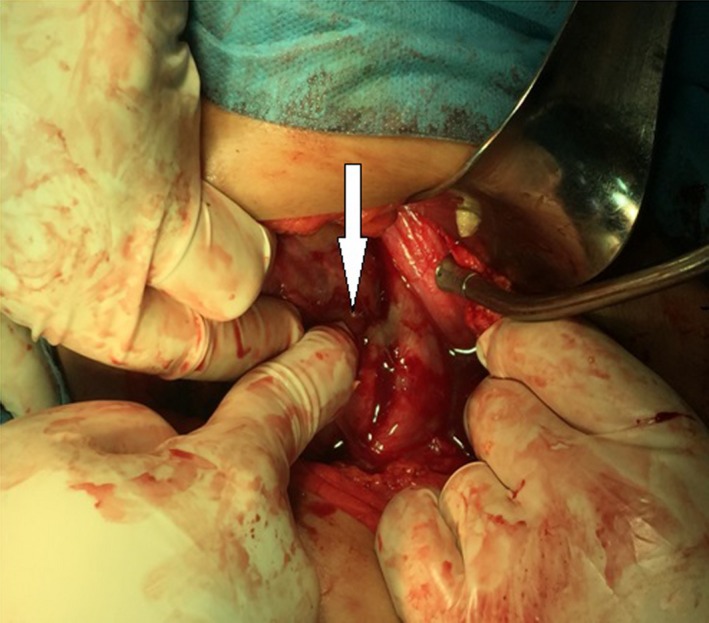
Intraoperative photo of the hydronephrotic hypoplastic left pelvic ectopic kidney. The abscess on the upper pole is shown by the arrow

**Figure 4:**
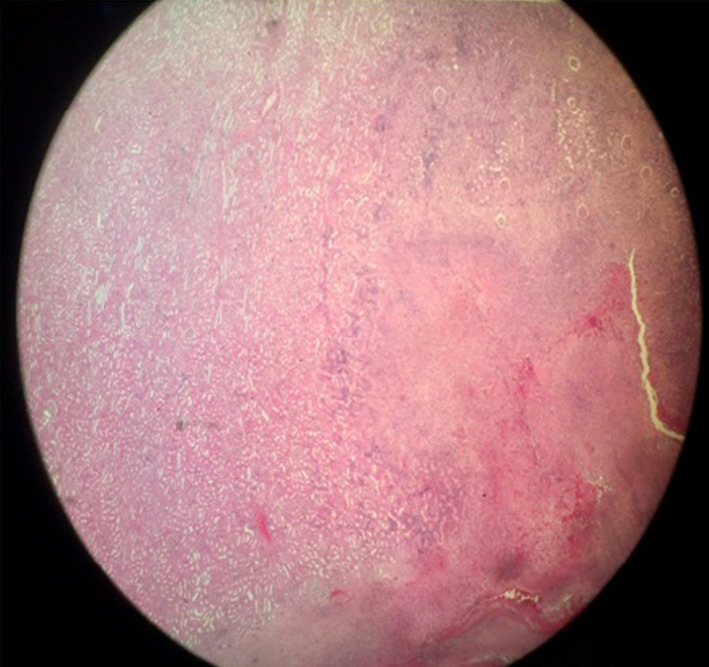
The microscopic examination of the specimen revealed areas of multiple necroses due to the abscesses and destruction of the normal renal parenchyma

A 22‐year‐old female, sexually active, patient referred to our department with fever and abdominal pain. The patient referred an obscure history of recurrent urinary tract infections. Palpation during clinical examination revealed acute pain of the left flank reflecting to the unilateral abdominal region. The patient presented high fever up to 39°C. Patient's history included left renal pelvic ectopia, and autoimmune hepatitis, which both diagnosed 8 years ago. During all this period, prezolon 10 mg x2 and azathioprine 50 x3 were administrated systematically. Leucocytosis with left turn of the type with predominance of neutrophils was the main finding in blood examinations. The urine analysis revealed increased white blood cells in the sample (100‐120 ps). Further examination with abdominal ultrasound and CT scan revealed an hypoplastic left pelvic kidney without hydronephrosis but with multiple abscesses in its upper pole (Figures 1 and 2). The patient treated conservatively with antibiotics. Ciprofloxacin (400 mg twice a day) and metronidazole (500 mg every 8 hours) were administrated intravenously. Although an initial temporary improvement, the disease progressed leading to an inevitable surgically performed left transabdominal nephrectomy. Macroscopical histological examination of the removed specimen revealed multiple abscesses in the upper pole of the kidney (Figure 3). Microscopical examination of the specimen confirmed the presence of multiple necrotic abscesses and the destruction of the normal renal parenchyma (Figure 4).

## 3 DISCUSSION

Ectopic kidney can be found in various sites including pelvis, iliac fossa, abdomen, thoracic cavity, and in some others more rarely. In our case, the ectopic kidney had an ipsilateral retroperitoneal location in pelvis. The pelvic kidney was located opposite to the sacrum below to aortic bifurcation. Autopsy studies reveal no significant difference between sexes. Several clinical studies suggest that renal ectopia is more often diagnosed in females. This is mainly due to the fact that women are submitted to detail uroradiologic examinations for urinary tract infections or associated genital anomalies more frequently.^5^

Pelvic ectopia's incidence has been estimated to occur in 1:2100 to 3000 autopsies while a small predominance in the left side.^6^ Embryologically, the ureteral bud arises from the Wolffian duct at the end of the 4th week of gestation and then grows cranially and acquires a cap of metanephric blastemal by the end of the 5th week. It is during this migration that the upper ureteral bud matures into a normal collecting system and medial rotation of the renal pelvis takes place. Factors that may prevent the orderly movement of the kidney include ureteral bud abnormal development, defective metanephric tissue, genetic abnormalities, maternal illnesses, or teratogenic causes.^1^, ^7^, ^8^

Ectopic kidneys are usually smaller than normal. The renal pelvis is usually anterior to the parenchyma because it has incompletely rotated. As a result, 56% of ectopic kidneys have a distended collecting system (hydronephrosis). Half of these cases result from obstructive at either at the ureteropelvic or the ureterovesical junction (70% and 30% respectively), 25% from reflux and 26% from malrotation alone.^4^ The origin and the course of both relative arteries and veins is probably unpredictable, and it usually depends on the location of the ectopic kidney.^9^

The majority of ectopic kidneys are clinically asymptomatic. Intermittent vague abdominal pain, ureteral colic due to obstruction, and urinary tract infections are the most frequent symptoms that may contribute to the correct diagnosis. Diagnostic algorithm includes ultrasonography, excretory urography, CT scan, radionuclide scanning, and retrograde pyelography. Arteriography may be helpful in defining the vascular supply in cases that demand surgical intervention. The ectopic kidney is not more susceptible to disease compared to a normal one except for development of hydronephrosis, calculus formation, and urinary tract infection.^4^ Furthermore; ectopic kidneys do not present increased risk for malignant transformation. Complicated urinary tract infection in ectopic kidney should be treated initially by conservative means. In most of the cases, appropriate antibiotic administration may be efficient. However, some cases may require surgical intervention including either placement of a double J‐stent ureteral catheter or open surgical exploration for the relief of obstruction due to the fact that urinary sepsis is a devastating possibility.

## 4 CONCLUSION

Although the present case does not add something new in the existing knowledge and practice posses significant educational interest and aims to point out some general practical principles. The clinical key message is included in the following sentences. Ectopic kidneys are usually asymptomatic. Intermittent vague abdominal pain, ureteral colic, and urinary tract infections may present nonspecific symptoms and set the suspicion. Imaging examinations are essential to the correct and accurate diagnosis. Treatment is usually conservative, and only in complicated cases, surgerical intervention is indicated.

## DISCLOSURE

Written informed consent was obtained from the patient for publication of this case report and any accompanying images.

## CONFLICT OF INTEREST

The authors declare no conflict of interest.

## AUTHORSHIP

SM, SA, and DF: contributed in patient's treatment, collected the material and the references, wrote and approved the manuscript. DT, IK, and PS: wrote and approved the manuscript.
